# SOCS1 is a critical checkpoint in immune homeostasis, inflammation and tumor immunity

**DOI:** 10.3389/fimmu.2024.1419951

**Published:** 2024-06-14

**Authors:** Grace M. Bidgood, Narelle Keating, Karen Doggett, Sandra E. Nicholson

**Affiliations:** ^1^ Inflammation Division, Walter and Eliza Hall Institute of Medical Research, Melbourne, VIC, Australia; ^2^ Department of Medical Biology, University of Melbourne, Melbourne, VIC, Australia

**Keywords:** SOCS1, cytokine, JAK-STAT, immunotherapy, autoimmunity, cancer

## Abstract

The Suppressor of Cytokine Signaling (SOCS) family proteins are important negative regulators of cytokine signaling. SOCS1 is the prototypical member of the SOCS family and functions in a classic negative-feedback loop to inhibit signaling in response to interferon, interleukin-12 and interleukin-2 family cytokines. These cytokines have a critical role in orchestrating our immune defence against viral pathogens and cancer. The ability of SOCS1 to limit cytokine signaling positions it as an important immune checkpoint, as evidenced by the detection of detrimental *SOCS1* variants in patients with cytokine-driven inflammatory and autoimmune disease. SOCS1 has also emerged as a key checkpoint that restricts anti-tumor immunity, playing both a tumor intrinsic role and impacting the ability of various immune cells to mount an effective anti-tumor response. In this review, we describe the mechanism of SOCS1 action, focusing on the role of SOCS1 in autoimmunity and cancer, and discuss the potential for new SOCS1-directed cancer therapies that could be used to enhance adoptive immunotherapy and immune checkpoint blockade.

## 1 Introduction

### 1.1 Cytokine signaling

**Figure 1:**
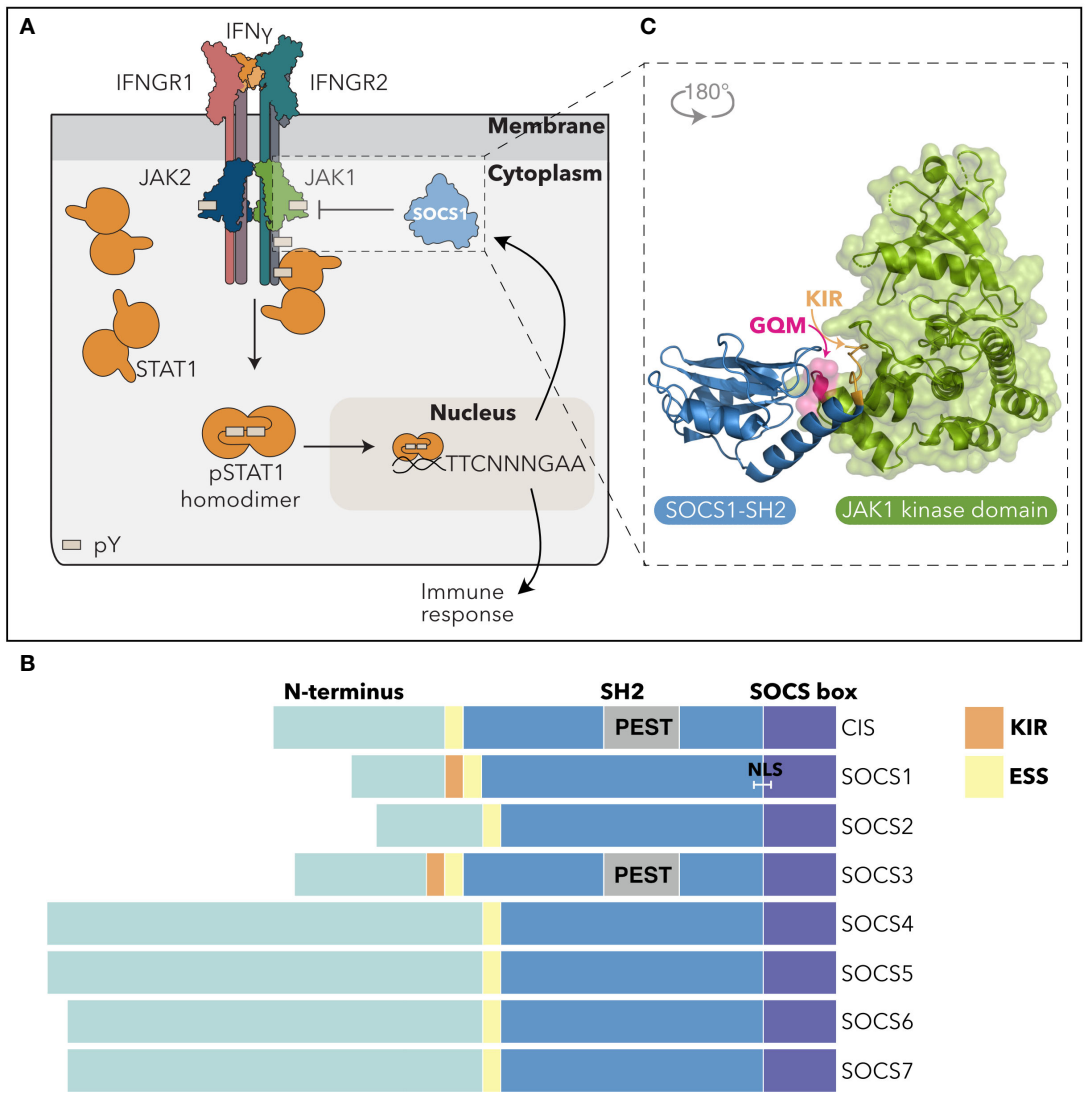
SOCS1 inhibits the IFNγ-driven JAK-STAT pathway. **(A)** SOCS1 negatively regulates IFNγ signaling. Upon engagement with the IFNGR complex, IFNγ induces tyrosine-phosphorylation and activation of JAK1 and JAK2. Activated JAK1/2 phosphorylate intracellular Tyr motifs in the IFNGR1, leading to STAT1 recruitment via the STAT1-SH2 domain and its subsequent phosphorylation by JAK. pSTAT1 dimers undergo a conformation change and translocate to the nucleus, binding to gamma interferon activation sites (GAS) to regulate transcription and drive a cellular response. SOCS1 is an IFNγ-response gene and is induced to inhibit IFNγ signaling in a classic negative feedback loop. pY = phosphotyrosine; TTCNNNGAA = IFNγ-activated promoter sequences. **(B)** SOCS domain architecture. The SOCS domain architecture consists of an unstructured N-terminal region of variable length (teal), a central SH2 domain (blue) and a C-terminal SOCS box motif (purple). SOCS1 and SOCS3 are distinguished by a KIR that precedes the ESS and SH2 domain region. The CIS and SOCS3 SH2 domains contain a PEST insertion (grey), while SOCS1 contains a putative NLS. **(C)** SOCS1 inhibition of JAK. Non-canonical binding of the SOCS1-SH2 domain (blue) to the JAK-GQM motif (pink), enables blocking of JAK-enzymatic activity via the SOCS1-KIR (orange). PDB: 6C7Y (5). SH2, Src-Homology 2 domain; ESS, Extended SH2 Sequence; KIR, Kinase Inhibitory Region; PEST, sequence rich in proline (P), glutamic acid (E), serine (S), and threonine (T); NLS*, nuclear localisation signal.

Cytokines are critical regulators of numerous cellular functions, including cell survival, proliferation, differentiation and chemotaxis, and are essential for growth, haematopoiesis, and innate and adaptive immunity. Cytokines direct cellular responses by binding to membrane-bound receptor complexes and activating the intracellular JAK-STAT (JAnus Kinase-Signal Transducers and Activators of Transcription) signaling pathway. The four mammalian JAK tyrosine kinases (JAK1, JAK2, JAK3 and TYK2) are constitutively associated with various receptor intracellular domains and become activated via trans or autophosphorylation of the JAK activation loop following cytokine engagement and receptor oligomerization. Activated JAKs then phosphorylate tyrosine residues within the receptor cytoplasmic domains, providing a docking site for Src homology 2 (SH2) domain-containing proteins, including the STATs (1). There are seven mammalian STAT proteins, STAT1–4, STAT5A, STAT5B, and STAT6 that dock to the receptors via their SH2 domains. The receptor associated JAKs then phosphorylate tyrosine residues in the STAT proteins, inducing a conformational change in pre-existing STAT dimers, and enabling nuclear translocation and a transcriptional response (2–4) (**Figure 1A**). In addition to the STATs, cytokines activate various other pathways, notably the PI3K/AKT (6) and Ras/MAPK (7) signaling cascades. Many cytokines are pleiotropic and redundant in nature, however, by activating various receptor-JAK-STAT combinations, individual cytokines can direct discrete cellular responses.

Excessive cytokine signaling can lead to inflammation and myeloproliferative disease, often with disastrous consequences, and signaling is therefore tightly regulated to maintain an appropriate cellular and systemic response. The JAK-STAT pathway is regulated at multiple levels, including by receptor trafficking, phosphatases, Protein-inhibitors of Activated STAT (PIAS) proteins, and the Suppressor Of Cytokine Signaling (SOCS) proteins (8–11).

### 1.2 SOCS family proteins

The SOCS protein family consists of eight proteins, CIS (Cytokine-Inducible SH2-containing protein) and SOCS1–7, that are characterized by an N-terminal region of varying length, a central Src homology 2 (SH2) domain and a C-terminal SOCS box (**Figure 1B**). CIS was discovered in 1995 by Yoshimura and colleagues as a regulator of interleukin (IL)-3 and erythropoietin (EPO) signaling (12), while SOCS1 was independently discovered in 1997 by three different groups through its capacity to suppress IL-6 signaling [SOCS1 (13)], recognition by an anti-STAT3 antibody [SSI-1 (14)], and binding to the JAK2 kinase domain [JAB (15)]. The homology between CIS and SOCS1 led to the discovery of the SOCS and greater SOCS box family via a conserved C-terminal motif referred to as the “SOCS box” (9).

The SOCS N-terminal regions are predicted to be unstructured (16) and vary in length and sequence, effectively dividing the SOCS proteins into those with a short (CIS, SOCS1–3) or long (SOCS4–7) N-terminal region (**Figure 1B**). To date, there are no clear functional roles for the N-terminal regions, as at least in structure-function studies, deletion of the N-terminus has no impact on SOCS function (17–19).

As in other SH2-containing proteins, the SOCS-SH2 domains recognise linear phosphotyrosine (pTyr) motifs within their target proteins, with binding selectivity dictated by residues flanking the pTyr and with a preference for a hydrophobic residue in the +3 position relative to the Tyr (5, 20–23). Structurally, the SOCS-SH2 domain displays the canonical SH2-fold of three central β-sheets flanked by two α-helices (22, 24). However, there are a few distinguishing features, most notably an additional α-helix termed the extended SH2 subdomain (ESS) located N-terminal to the conserved SH2 sequence. The ESS interacts with residues either side of the SOCS-pTyr-binding (BC) loop, stabilizing the interaction with pTyr (20, 25). In addition, the CIS and SOCS3-SH2 domains contain an unstructured PEST motif insertion (rich in proline, glutamic acid, serine and threonine) that at least in SOCS3, appears to regulate protein stability (20).

SOCS1 and SOCS3 have a unique ability to directly inhibit JAK enzymatic activity. This was first demonstrated by Endo and colleagues for SOCS1 (15), with the activity subsequently shown to be mediated by a short “kinase inhibitory region” or “KIR” that preceded the ESS and SH2 domain (19, 25). Structural and biophysical characterization of the SOCS: JAK complexes revealed a direct interaction between the SOCS1/3-ESS and BC loop and a GQM motif present in JAK1, JAK2 and TYK2 (but not JAK3) that enabled the SOCS-KIR to act as a pseudosubstrate, partially blocking the substrate binding groove on JAK and subsequent JAK enzymatic activity (5, 26) (**Figure 1C**). The SOCS1-SH2 domain is thought to interact with the phosphorylated JAK activation loop (25), and at least under some conditions, SH2 interaction with phosphorylated JAK is required for inhibition of kinase activity (27). However, given the structural constraints it seems unlikely that SOCS1 can simultaneously interact with a single JAK molecule via both the substrate binding groove and the activation loop (5), thus raising some interesting questions about the stoichiometry of the SOCS1:JAK complexes that are yet to be resolved.

The SOCS box contains two motifs, the BC box and Cul5 box that interact respectively, with the adaptor proteins Elongin B and C, and the E3 ubiquitin ligase scaffold protein Cullin-5. Together with Ring Box 2 (RBX2), this forms an E3 ligase complex that mediates the ubiquitination and proteasomal degradation of SH2-bound targets (28–30). The SOCS proteins therefore function as substrate receptors for a Cullin-5 RING ligase (CRL5) complex. However, Cullin-5 interaction with the SOCS1 and SOCS3-SOCS boxes is relatively weak compared to other SOCS family members (100 and 10-fold lower affinity, respectively) (31), suggesting that SOCS1 and SOCS3 predominantly act as negative regulators through their ability to directly inhibit JAK catalytic activity (5).

Finally, SOCS1 has been reported to have a nuclear localization signal (NLS) sequence that bridges the SH2 domain and SOCS box, consistent with various reports that place SOCS1 in the nucleus (32–34). However, how nuclear localization impacts SOCS1 regulation of cytokine signaling remains unclear.

### 1.3 Suppressor Of Cytokine Signaling 1

SOCS1 is a critical negative regulator of signaling in response to type I, II and III IFN and IL-2, IL-4, IL-7, IL-12, IL-13, IL-15, and IL-21 (35–43). SOCS1 is constitutively expressed in the thymus where it plays a vital role in T cell development and homeostasis (44–46). However, under infectious challenge or during inflammatory disease, SOCS1 expression is induced in response to various cytokines, including those known to be regulated by SOCS1, enabling it to act in a classic negative feedback loop (**Table 1**).

**Table 1:** SOCS1 induction in response to various cytokines.

Cytokine	JAKS	Cell type		SOCS1 detection*	Reference
		Human	Rodent		
JAK/STAT cytokines that induce SOCS1 and are regulated by SOCS1 ^#^					
IFNα/β	JAK1 TYK2	Huh-7, A-357, HT-144 cells; epidermal melanocytes	BMDMs	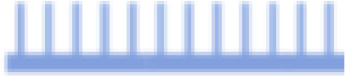 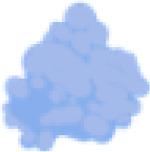	(35, 37, 47–49)
IFNλ	JAK1 TYK2	Huh-7, A-549 cells		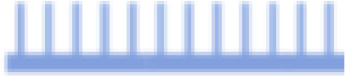 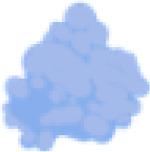	(37, 48, 50)
IFNγ	JAK1 JAK2	Huh-7 cells	BM; BMDMs; MEFs; colonic epithelial cells	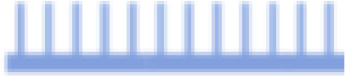 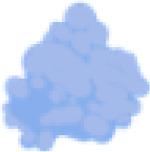	(13, 35, 38, 39, 48, 49)
IL-2	JAK1 JAK3	T cells	T cells	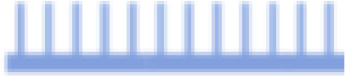 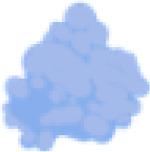	(41, 42, 51)
IL-4	JAK1 JAK3	Osteoarthritic chondrocytes; A-549, U-937 cells	BMDMs; T cells; MEFs; colonic epithelial cells; CT.4S cells	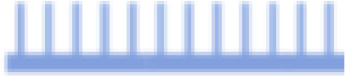 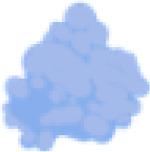	(14, 39, 52–56)
IL-7	JAK1 JAK3	CD8^+^ T cells; B cells	CD8^+^ T cells; B cells; T cells	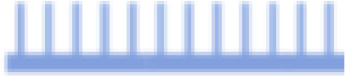 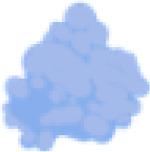	(42, 44, 57, 58)
IL-12	JAK2 TYK2		T cells; BMDCs	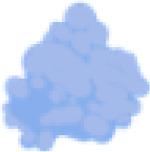	(36, 59)
IL-13	JAK1 TYK2	U-937 cells	TGMBE-02–3 cells; MEFs; BM; lung	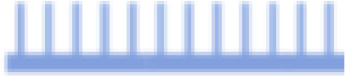 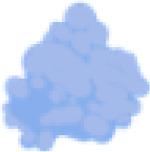	(13, 43, 52, 56)
IL-15	JAK1 JAK3		CD8^+^ T cells; T cells	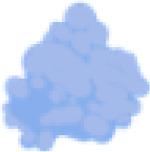	(40–42)
IL-21	JAK1 JAK3	CD8^+^ T cells; DCs	CD8^+^ T cells	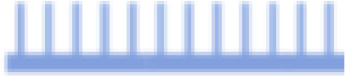 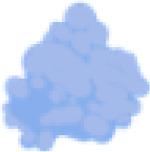	(60–62)
JAK/STAT cytokines that induce SOCS1 but are not known to be regulated by SOCS1					
EPO	JAK2		BM; 32D, HCD-57 cells	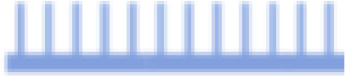	(13, 15)
G-CSF	JAK2		NFS-60 cells	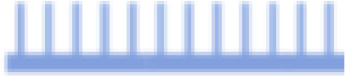	(14)
GM-CSF	JAK2		BM	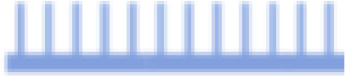	(13)
GH	JAK2		3T3-F442A cells	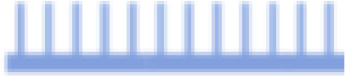	(63)
IL-3	JAK2		BM	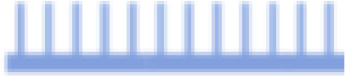	(13)
IL-6	JAK1 JAK2		CD4^+^ T cells; BMDMs; liver; MH60.BSF2 and M1 cells	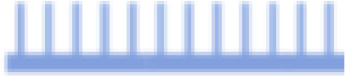 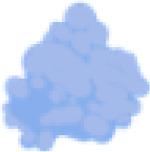	(13–15, 64)
IL-23	JAK2 TYK2	T cells		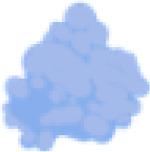	(65)
IL-27	JAK1 JAK2	CD8^+^ and CD4^+^ T cells		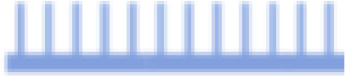 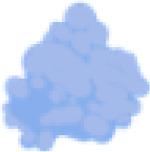	(66–68)
LIF	JAK1 JAK2		M1 cells	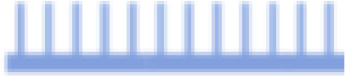	(14)
PRL	JAK2	T-47D cells	Liver	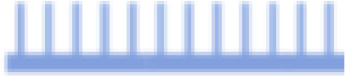	(69)
TSH	JAK1 JAK2	Endometrial stromal cells	FRTL-5 cells	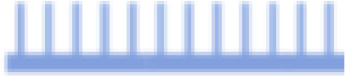 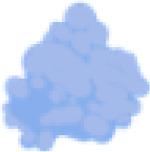	(70, 71)

* indicates detection of SOCS1 mRNA 
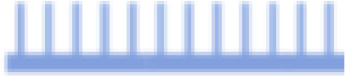
 or protein 
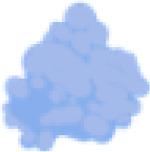
.

**^#^** as evidenced by SOCS1-deficient cells.

MEFs, mouse embryonic fibroblasts; BM, bone marrow; BMDMs, bone marrow-derived macrophages; DCs, dendritic cells.

The importance of SOCS1 in limiting spontaneous inflammation is evident from mouse models, where homozygous deletion of the *Socs1* gene (*Socs1^-/-^*) results in perinatal lethality around 3-weeks of age, due to fatty degeneration and necrosis of the liver, and immune infiltration of multiple organs (46, 72). The multi-organ inflammation and lethality results from hyperresponsiveness to inflammatory cytokines and can be rescued by compound deletion of *Ifng* or treatment with neutralising anti-IFNγ antibodies (38, 45, 73). Mice lacking both *Socs1* and *Ifng* eventually develop a fatal inflammatory disease at around 6-months of age, indicating the involvement of other cytokine pathways (74). *Socs1^-/-^* lethality is similarly rescued by deletion of *Rag2* (no T, B or NK cells), *Stat6* (IL-4) or components of type I IFN signaling, IFNAR1 and TYK2 (35, 45, 47, 75). Lethality is also partially rescued by deletion of *Stat4* (IL-12), with mice succumbing to inflammatory disease at 1–2 months of age (36).

In addition to an enhanced response to IFNγ, the pathology in *Socs1^-/-^* neonates is associated with elevated serum levels of IFNγ, most likely due to excessive production by T cells in response to IL-1, IL-2 and IL-12 (36, 45, 75, 76). The inflammatory disease is no doubt compounded by a deficiency in peripheral Foxp3^+^ regulatory and γδ T cells in the *Socs1^-/-^* mice (77, 78). Additionally, SOCS1 has been shown to limit dendritic cell (DC) maturation and antigen-presentation through inhibition of IFNγ and IL-4 (79, 80).

The KIR and SH2 binding to pTyr are both required for SOCS1 to inhibit signaling; mice bearing single point mutations in either domain (KIR:F59A; SH2:R105A) phenocopy full *Socs1* deletion, dying shortly after birth (27). In contrast, mice lacking the SOCS1-SOCS box survive, although they develop a multi-organ inflammatory disease with age (81). This is consistent with weak Cullin-5 interaction with the SOCS1-SOCS box (31), and further evidence that SOCS1 does not rely on its E3 ubiquitin ligase activity to inhibit cytokine signaling.

The apparent selective regulation of IFN, IL-12 and IL-2 family signaling by SOCS1 is intriguing. SOCS1 directly inhibits JAK1, JAK2 and TYK2 activity through the KIR, and the SOCS1-SH2 domain has been proposed to interact with the phosphorylated activation loops of all four JAK catalytic domains (5, 25). Given that various combinations of JAK1, JAK2 and TYK2 are associated with most cytokine receptors (either singly or in combination), it is unclear how SOCS1 selectively regulates IFN, IL-12 and IL-2 family signaling, although this may simply reflect greater induction of SOCS1 protein by these cytokines (**Table 1**). To date, no receptor pTyr residues have been identified as high affinity SOCS1-SH2 binders, despite pTyr interaction being required for SOCS1 inhibition of signaling (5, 27). In contrast, SOCS3 binds with high affinity to phosphorylated tyrosines within the gp130, IL-12, leptin and G-CSF receptors (82–87), providing a clear mechanism underlying the selective SOCS3 regulation of the corresponding cytokine pathways.

In addition to being induced by JAK/STAT signaling, SOCS1 expression is down-regulated by a well-characterised microRNA, miR-155. MiR-155 is derived from a non-coding transcript referred to as the B cell integration cluster and is upregulated in various immune cells in response to inflammatory stimuli and infection, with multiple targets in addition to *SOCS1* [reviewed in (88, 89)]. An elegant study by Lu and colleagues (90) mutated the miR-155 binding site in the *Socs1* mRNA 3’UTR, partially recapitulating the effects of miR-155 deletion to reduce disease severity in the autoimmune encephalomyelitis (EAE) mouse model of multiple sclerosis, and limit the expansion of NK cells in response to murine cytomegalovirus (MCMV) infection (90).

## 2 SOCS1 in human disease

### 2.1 SOCS1 in autoimmunity/inflammatory disease

Cytokines and inflammatory mediators are strongly implicated in the pathogenesis of autoimmune diseases. For example, type I and type II IFN (91, 92), and IL-12 (93) are associated with systemic lupus erythematosus (SLE), and the IL-12-driven transcription factor *STAT4* is a dominant genetic risk allele (93, 94). Exacerbated IFNγ and IL-12 responses are also known to drive chronic joint inflammation in rheumatoid arthritis (95–97). Importantly, SOCS1 inhibits the cellular response to these key inflammatory mediators of SLE and RA.

**Figure 2:**
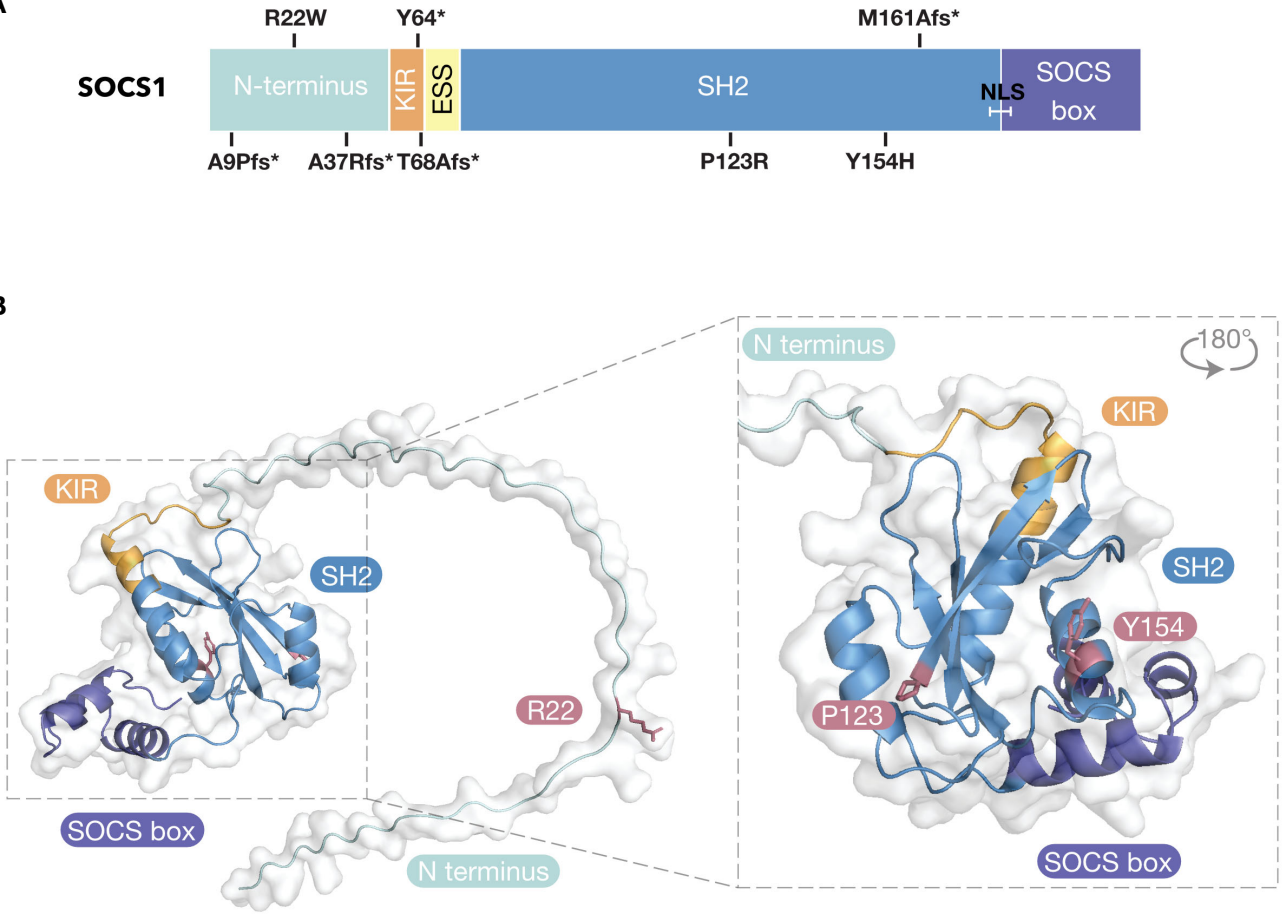
Inborn Errors of Immunity of SOCS1. **(A)** SOCS1 variants mapped to domain architecture. * = stop codon; fs, frameshift; SH2, Src-Homology 2; KIR, Kinase Inhibitory Region; ESS, Extended SH2 Sequence; NLS, Nuclear Localization Sequence. **(B)** SOCS1 missense variants mapped to predicted structure of SOCS1 (AlphaFold 2.0 database).

Inborn errors of immunity arising in *SOCS1* have been reported in patients presenting with a range of clinical phenotypes, including immune dysregulation with multi-system autoimmunity, autoimmune diseases such as SLE and chronic autoimmune cytopenia, and malignancy (98–104). The majority of reported inborn errors of immunity in *SOCS1* are private (not reported in the general population), heterozygous, autosomal dominant and loss-of-function variants (98–100, 102, 103) (**Figure 2**). The lack of homozygosity implies the *SOCS1* gene is indispensable, consistent with the lethal phenotype of *Socs1^-/-^* mice (46, 72). Given the majority of variants result in loss of protein, it implies the dose of SOCS1 is important, accounting for the autosomal dominance and consistent with the inflammatory disease in aging *Socs1^+/-^* mice (105). Two rare *SOCS1* missense variants have also been reported in *cis* (P50L, A76G), however, they appeared to have little impact on SOCS1 function (104).

Patient mutations within the SOCS1-KIR (Y64>stop, T68A with a frameshift resulting in a stop codon: fs>stop) and the SH2 domain (M161Afs>stop, P123R, Y154H) result in loss of SOCS1 expression. The two unrelated patients that harbored the Y64>stop variant presented with Hyper IgE-like syndrome with eczema (P1) and eosinophilic allergic alveolitis (P2) (98). The patient carrying the T68>fs variant presented with severe dermatitis, recurrent skin infections and psoriatic arthritis (P3) (99). P4 and P5 carried the M161A frameshift variant (M161Afs>stop) and were from unrelated families. P4 presented with common variable immunodeficiency (CVID)-like phenotype (98) and P5 presented with Evans syndrome (100). Four patients within the same family were heterozygous for Y154H and presented with a range of autoimmune diseases including SLE (P6), immune thrombocytopenia (ITP; P7), psoriasis (P8 and P9) and spondyloarthritis, autoimmune hepatitis and pancreatitis (P9) (100). Two patients within the same family were heterozygous for P123R and presented with severe ITP (P10) and ITP thyroiditis polyarthritis (P11) (100). The remaining five patients (P12–17) carried *SOCS1* variants that clustered within the N-terminal domain (A9Pfs>stop, R22W, A37Rfs>stop) (100, 102). These patients suffered from various autoimmune diseases, including Evans syndrome, coeliac disease, psoriasis, SLE, and multisystem inflammatory syndrome in children (MIS-C). While the KIR and SH2 missense variants likely disrupted the SOCS1 structure (thus resulting in loss of SOCS1 expression), the R22W variant may be the first evidence that the N-terminal region of SOCS1 is required for SOCS1 inhibitory activity (**Figure 2**).

For diseases such as SLE and eosinophilia, where the key cytokines driving pathology are known [type I IFN (106); IL-2 family cytokines (107), respectively], it is clear how loss of SOCS1 results in pathogenesis. Similarly, the dermatitis seen in P3 only responded to treatment with dual IL4Rα and IL17A blockade (99), making an obvious link to SOCS1 regulation of the IL-4 pathway. However, diseases such as CVID and Evans syndrome have unknown causes and the patient’s pathology arises from dysregulation of multiple immune pathways, as do other inborn errors of immunity, such as *STAT1* gain-of-function variants. In addition, some family members harboring *SOCS1* variants were asymptomatic, indicating *SOCS1* haploinsufficiency does not have complete clinical penetrance. Despite this, *SOCS1* haploinsufficiency has complete cellular penetrance. Cells derived from all individuals with loss-of-function SOCS1 variants consistently had augmented IFNγ, IL-2 and IL-4 signaling responses, with increased STAT1, STAT5 and STAT6 phosphorylation, respectively (98–103). Consequently, the etiology of *SOCS1* haploinsufficiency still remains unclear (100). Current treatment of *SOCS1* haploinsufficient patients largely relies on immunosuppressive drugs such as corticosteroids or JAK-inhibitors to lower systemic inflammation (98–103), however, given the potential side-effects associated with long-term treatment, more targeted approaches are required.

### 2.2 SOCS1 in cancer

It is evident that SOCS1 inhibition of cytokine signaling is required to maintain immune homeostasis and resolve inflammatory signaling. Given the importance of inflammation and the immune response in cancer initiation and development (108), and the contribution of hyperactive JAKs to myeloproliferative disorders and lymphoid cancers (109), it is not surprising that a growing body of work has highlighted a role for SOCS1 in cancer.

### 2.3 SOCS1 is linked to tumor suppression

In many cases, silencing of the *SOCS1* gene and/or reduced mRNA or protein levels, is associated with cancer versus normal tissue (110–112). Hypermethylation of the *SOCS1* gene has been reported in multiple cancers and has been linked to cell growth in hepatocellular, pancreatic, oesophageal and gastric cancer (113–116). For instance, in hepatocellular carcinoma, hypermethylation of the *SOCS1* promoter region was observed in 65% of patient samples, with restoration of SOCS1 levels inhibiting cell growth *in vitro* (113, 117). In addition to gene silencing, high levels of microRNAs such as miR-19a/b or miR-155 correlated with low *SOCS1* expression in cancer cell lines and primary tumors (118–122). Although miRNAs have many targets, Jiang et al. (120), demonstrated that *SOCS1* siRNA recapitulated the oncogenic properties of miR-155 in breast cancer, while restoration of SOCS1 attenuated the impact of miR-155.

Several somatic *SOCS1* mutations have been associated with B cell lymphomas, such as Diffuse large B cell lymphoma (DBCL) and Hodgkin lymphoma (123–125). Detrimental mutations were associated with the germinal centre B (GCB) cell-like subtype of DBCL (123), consistent with IL-4-JAK-STAT6 signaling in this subtype (126) being regulated by SOCS1. In addition, a patient with an inborn error of immunity in *SOCS1* (A9P>fs) also presented with Hodgkin lymphoma (as well as psoriasis and Coeliac disease) (100).

A polymorphism in the SOCS1 promoter region (-1478 CA>del) has been reported to increase *SOCS1* expression in atopic asthma (127), and has been associated with breast and gastric cancer, as well as with a poorer outcome in colon cancer (128–131). The effect of this polymorphism remains unclear, although it does not appear to be linked to tumor suppression.

Although some of the growth inhibitory effects of SOCS1 in cancer can be attributed to regulation of JAK/STAT signaling, SOCS1 has also been reported to target other pathways, including via MET receptor tyrosine kinase, p21, p53 and NF-kB (132–139). Consistent with its nuclear localization, SOCS1 interaction with the p53 tumor suppressor has been proposed to promote p53 activation and cellular senescence (136, 137). In addition, SOCS1-mediated ubiquitination and degradation of the NF-kB p65 subunit limits NF-kB-driven transcription (138, 139). Hence reduced SOCS1 levels would potentially limit p53 activity and potentiate NF-kB-driven cell growth.

The concept of SOCS1 as a tumor suppressor is reviewed in more detail by Ilangumaran and colleagues (this research topic) (140). The remainder of this review will focus on SOCS1 regulation of anti-tumor immunity.

### 2.4 The IFNγ-SOCS1 axis and immune checkpoint blockade

Type II IFN (IFNγ) and to a lesser extent the type I IFNs, drive a transcriptional program in cancer cells that results in apoptotic cell death, in addition to influencing anti-tumor immunity. This includes secretion of chemoattractants, activation of cytotoxic T cells and enhancement of antigen presentation (141–148). However, IFNγ also drives expression of molecules on cancer cells such as programmed death-ligand (PD-L)1 and PD-L2 that inhibit T cell function, contributing to immune evasion (149). The impact of these opposing effects is likely to depend on the strength and persistence of signaling, as well as the cancer context and tumor microenvironment (TME).

Antibody therapies that target the immune checkpoints PD-L1/PD1 and CTLA4 have revolutionized cancer treatment (immune checkpoint blockade; ICB) (150). However, many patients remain refractory to treatment or develop resistance (151). The importance of the IFNγ pathway (and its regulation by SOCS1) was established by a series of seminal studies that used genetic screens and analysis of patient samples to show that defects in IFNγ pathway components (*Ifngr1, Ifngr2, Jak1, Jak2, Stat1, Irf1*) or amplification of the negative regulators *Socs1* and *PIAS4*, conferred resistance to ICB (152–157).

Consistent with this, depletion of *Socs1* in a lung cancer cell line that was resistant to ICB, restored the IFNγ response and sensitized tumours to anti-PD1 therapy (158). In another study, Dhainaut and colleagues (159) used spatial transcriptomics coupled with functional genomics to understand the impact of individual genes within tumor lesions. Although loss of *Socs1* increased CD4^+^ and CD8^+^ T cell infiltration into the tumor lesions, it also promoted tumor growth, likely via IFNγ-driven expression of PD-L1 inhibiting T cell function. Critically, anti-PD-L1 blockade preferentially depleted *Socs1* deficient lesions (159).

A study by Song et al. (160), linked cancer-specific defects in microRNA processing to reduced miR-155 levels, with a corresponding increase in SOCS1 that suppressed IFNγ responses and increased resistance to T cell killing. This was associated with decreased PD-L1, antigen presentation and secretion of the T cell-attractant chemokines CXCL9 and CXCL10 (160). In a second study, high miR-155 expression in tumors correlated with improved anti-tumor immune profiles and outcomes in breast cancer patients. This was connected to miR-155 suppression of SOCS1 enhancing IFN-driven CXCL9, CXCL10 and CXCL11 production and immune infiltration, sensitizing to ICB (161). More recently, House and colleagues (162) identified IRF-I as a key negative regulator of CXCL9 production in cancer and myeloid cells through induction of SOCS1 and subsequent inhibition of IFNγ signaling.

These studies highlighted a link between reduced SOCS1 and (i) increased expression of immune checkpoints, thus sensitizing tumors to ICB, and (ii) IFN-driven chemokine production that increased immune cell infiltration into the TME. Furthermore, they suggest that upregulation of PD-L1 in response to reduced SOCS1 levels could potentially improve patient responses to ICB.

### 2.5 Impact of SOCS1 in immune control of cancer

While global genetic deletion revealed the critical role of SOCS1 in immune cell development (42, 44, 45, 163), the gross defects in neonatal mice confounded the study of mature immune cell function, particularly in the context of tumor immunity. Subsequent studies have revealed a key inhibitory role for SOCS1 in multiple immune subsets that coordinate to mount an effective anti-tumor immune response.

A genome wide CRISPR screen identified SOCS1 as a negative regulator of proliferation and cytotoxicity in primary human T cells (164). *Socs1* was later identified as a non-redundant inhibitor of antigen-experienced CD4^+^ T cell proliferation and effector function in mice (165). Inactivation of *Socs1* in CD4^+^ T cells maintained proliferation, while inactivation in CD8^+^ T cells enhanced survival and effector function; adoptive transfer of *Socs1*-deficient CD4^+^ and CD8^+^ cells together giving greater therapeutic benefit. Similar results were obtained in human CD19-chimeric antigen receptor (CAR)-T cells, highlighting the unique roles of SOCS1 in CD4^+^ vs CD8^+^ T cells, and the synergistic potential of targeting *SOCS1* in adoptive CAR-T cell immunotherapy (165). A second genetic screen, this time in CD8^+^ cells, identified *Socs1* as a key checkpoint that not only restricted CD8^+^ T cell expansion, but also infiltration of CD8^+^ cells into the TME (166). The authors went on to evaluate *SOCS1* deletion in primary human T cells and tumor infiltrating lymphocytes, demonstrating enhanced IL-2-driven STAT5 and IL-12-STAT4 activation, associated with increased IFNγ production and reduced tumor growth in an immunodeficient mouse model (166).

SOCS1 expression in DCs restricted antigen presentation and the magnitude of the resulting adaptive immune response, with reduction of SOCS1 in DCs promoting T cell-mediated anti-tumor immunity (167–170). *Socs1^-/-^* DCs induced a stronger T helper (T_H_)1 response, associated with increased DC production of IFNγ (168). Conditional deletion of *Socs1* in myeloid cells resulted in a significantly reduced B16F10 tumor burden and suppressed DSS/DHM-induced colon cancer growth. In co-cultures, DCs derived from these mice induced higher IFNγ production from CD4^+^ T cells and enhanced CD8^+^ T cell cytotoxic activity (171). These studies highlight a cell-intrinsic role for SOCS1 in macrophages and DCs that restricts myeloid potentiation of the anti-tumor T cell response.

**Figure 3:**
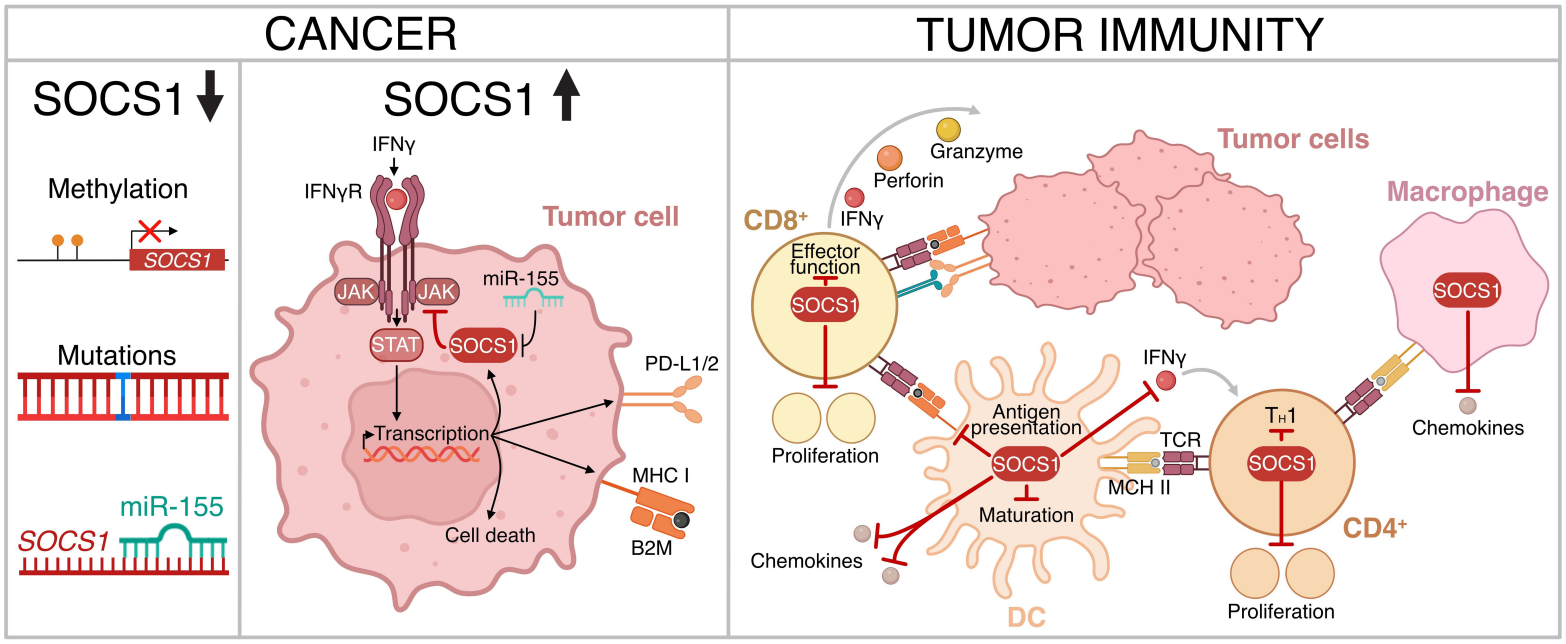
The role of SOCS1 in regulating tumor immunity. Downregulation of expression or loss of SOCS1 function has been observed in certain cancer subtypes (left panel). SOCS1 can confer resistance to immune checkpoint blockade via inhibition of IFNγ signaling and down-regulation of immune checkpoints PD-L1/2 (center panel). In addition, SOCS1 limits anti-tumor immunity through various immune cell types (right panel). Figure created with BioRender.com.

SOCS1 clearly has specific roles in CD4^+^ and CD8^+^ T cells, DCs and macrophages that limit anti-tumor immunity, in addition to a tumor intrinsic role that also impacts immune cell recognition and killing. Taken together, this positions SOCS1 as a potentially powerful negative regulator of the anti-tumor immune response (**Figure 3**).

## 3 Discussion

Cytokines such as the IFNs are an important aspect of our immune defence and are critical for the elimination of infectious pathogens and transformed or cancerous cells. As the key negative regulator of many of these pathways, SOCS1 is a bona-fide intracellular immune checkpoint, with loss of SOCS1 resulting in unrestrained cytokine responses associated with autoimmunity and other inflammatory diseases. Correspondingly, elevation of SOCS1 levels in both cancer and immune cells, limits the effectiveness of cytokines that drive anti-tumor immunity. Both scenarios present attractive opportunities for therapeutic intervention.

SOCS1 deficiency has been implicated in the pathogenesis of lupus, uveitis, and asthma, highlighting the role of SOCS1 in regulating immune homeostasis (52, 77, 172–176). Identifying individuals with inflammatory disease associated with genetic inactivation of *SOCS1* may help stratify patients for treatment with JAK inhibitors. SOCS1 has the potential to dampen signaling from all IFNs, in addition to IL-2 family cytokines and IL-12. This represents an overlapping spectrum of activities compared to the current JAK inhibitors which include JAK-specific and pan-inhibitors (177). Strategies that increase SOCS1 levels or mimic its activity to reduce cytokine signaling may have utility as an alternative to or in combination with JAK inhibitors and would have a broader spectrum of activity than biologics targeting single receptor chains or cytokines.

Cell penetrating-SOCS1 peptidomimetics that mimic the activity of the SOCS1 kinase inhibitory region (KIR) have successfully been used to treat lupus-like disease, uveitis, and experimental autoimmune encephalitis (model of multiple sclerosis) in rodent models (178, 179). Additionally, cell penetrating forms of recombinant SOCS1 protein have been shown to suppress cytokine signaling (180). These studies highlight the potential of enhancing SOCS1 levels and/or developing drugs that mimic SOCS1 activity in the treatment of autoimmune disease. Alternative approaches to increase SOCS1 levels, such as antagomirs that target miR-155, may also have some utility given the recent advances in RNA delivery systems (181).

SOCS1 has been reported to have both positive and negative effects in the context of cancer control. However, although reduced SOCS1 expression correlated with poor prognosis in a variety of solid cancers, in many instances the relationship between SOCS1 levels and cancer etiology remains unclear. In addition, a complex mix of driver and co-operating mutations together with the localised inflammatory milieux may result in varying SOCS1 levels. This is often further complicated by an inability to distinguish between intrinsic tumor expression of SOCS1 and expression in the surrounding cellular environment. The growing application of single cell sequencing and spatial omics should advance our understanding of how SOCS1 expression impacts disease progression in specific cancer and cellular contexts.

CRISPR-Cas9 gene editing in whole genome screening has been a powerful discovery-tool in many fields, as illustrated by the studies discussed in this review. One of the striking findings to emerge, in addition to the importance of IFNγ signaling in maintaining the response to ICB, is the potential synergy to be gained by reducing *SOCS1* in tumors in combination with ICB. SOCS1 is also a critical checkpoint in immune cells, limiting aspects of immune cell development as well as effector cell function. Interestingly, tumor-specific, and immune-cell reduction of SOCS1 both lead to increased chemokine secretion and lymphocyte recruitment and infiltration into the TME. Reducing SOCS1 levels to sustain IFNγ signaling may therefore be a useful therapeutic approach to reinvigorate lymphocyte function in an immunosuppressive TME.

However, it is challenging to target an intracellular protein, particularly one that is part of a closely related family and relies on a common modality of binding to pTyr for its mechanism of action. One interesting example of modulating SOCS1 levels, is the lipid nanoparticle (LNP) delivery of SOCS1-targeting small interfering RNA (siRNA) to bone marrow-derived DCs. Vaccination with OVA-specific DCs containing *Socs1* siRNA prior to challenge with OVA-bearing B16F10 melanoma cells, successfully suppressed tumor growth through increased cytokine secretion and antigen presentation (182).

The most obvious and perhaps feasible application for reduced SOCS1 levels is the field of adoptive T cell therapy. Adoptive T cell therapies genetically modify a patient’s T cells to express receptors for tumor antigens (CAR-T cells), prior to expansion and infusion back into the patient (183). CAR-T cells have shown impressive clinical efficacy in haematological malignancies, with six FDA-approved CARs now in the clinic. However, CAR-T cells are much less effective in solid tumors, most likely due to an immunosuppressive TME (184–189). An alternative approach involves expansion of a patient’s TILs prior to adoptive transfer, with the first TIL-therapy recently approved by the FDA for melanoma (lifileucel; Amtagvi, Iovance Biotherapeutics) (190). The studies discussed here highlight the potential for inactivation of *SOCS1* in CD8^+^ and/or CD4^+^ CAR-T cells or TILs, to overcome T cell exhaustion and improve proliferation, survival, and effector function in adoptive T cell immunotherapy. Going forward it will be critical to evaluate potential exacerbation of adverse effects, such as cytokine release syndrome, as a result of *SOCS1* deletion (191).

## 4 Concluding remarks

In summary, SOCS1 is an important immune checkpoint and an attractive target in both autoimmunity and cancer. Its intrinsic roles in both tumor and immune cells make it a central player that restricts IFNγ-driven killing and facilitates escape from PD1/CTLA4 blockade. The challenge will be to find innovative ways to target SOCS1 in the different disease contexts. We look forward to watching the next chapter unfold with interest.

## Author contributions

GMB: Data curation, Writing – original draft, Writing – review & editing. NK: Writing – original draft, Writing – review & editing. KD: Supervision, Writing – review & editing. SEN: Conceptualization, Funding acquisition, Supervision, Writing – review & editing.
